# Supplementary material for: Loss of muscle PDH induces lactic acidosis and adaptive anaplerotic compensation via pyruvate-alanine cycling and glutaminolysis
Source: J Biol Chem. 2023 Oct 20;299(12):105375. doi: 10.1016/j.jbc.2023.105375 (PMC10692893; doi:10.1016/j.jbc.2023.105375)
Supplement: Supplemental Figs. S1 and S2, and Table S1 [file mmc1.pdf]

Loss of Muscle PDH Induces Lactic Acidosis and Adaptive Anaplerotic Compensation via Pyruvate-Alanine Cycling and Glutaminolysis

Keshav Gopal[1,2,3†], Abdualrahman Mohammed Abdualkader[4,5,6†], Xiaobei Li[4,5,6], Amanda A. Greenwell[1,2,3], Qutuba G. Karwi[2,3,7], Tariq R. Altamimi[2,3], Christina Saed[1,2,3], Golam M. Uddin[2,3], Ahmed M. Darwesh[1,3], K Lockhart Jamieson[1,3], Ryekjang Kim[1,2,3], Farah Eaton[1,2,3], John M. Seubert[1,3], Gary D. Lopaschuk[2,3], John R. Ussher[1,2,3], Rami Al Batran[4,5,6,*]

[1]Faculty of Pharmacy and Pharmaceutical Sciences, University of Alberta, Edmonton, AB, Canada
[2]Alberta Diabetes Institute, University of Alberta, Edmonton, AB, Canada
[3]Cardiovascular Research Centre, University of Alberta, Edmonton, AB, Canada
[4]Faculty of Pharmacy, Université de Montréal, Montréal, QC, Canada
[5]Montreal Diabetes Research Center, Montréal, QC, Canada
[6]Cardiometabolic Health, Diabetes and Obesity Research Network, Montréal, QC, Canada
[7]Division of BioMedical Sciences, Faculty of Medicine, Memorial University of Newfoundland, Saint John's, NL, Canada

[†]Equal contribution

[*]Address for correspondence:
Dr. Rami Al Batran,
Bureau 3216, Pavillon Jean-Coutu
2940, Chemin de la Polytechnique
Faculty of Pharmacy
Université de Montréal
Montréal, QC H3T 1J4
Canada

Tel: 001 514 343- 6111 Ext 14500
Email: rami.al.batran@umontreal.ca

Supporting information

Supporting Figure S1. Skeletal muscle PDH deficiency induces lactic acidosis, pyruvate-alanine cycling, and glutaminolysis.

Supporting Figure S2. HFD supplementation abolishes overt phenotype induced by muscle PDH deficiency.

Supporting Table S1. Primer sequences for qPCR analysis.

Supporting Figure S1.

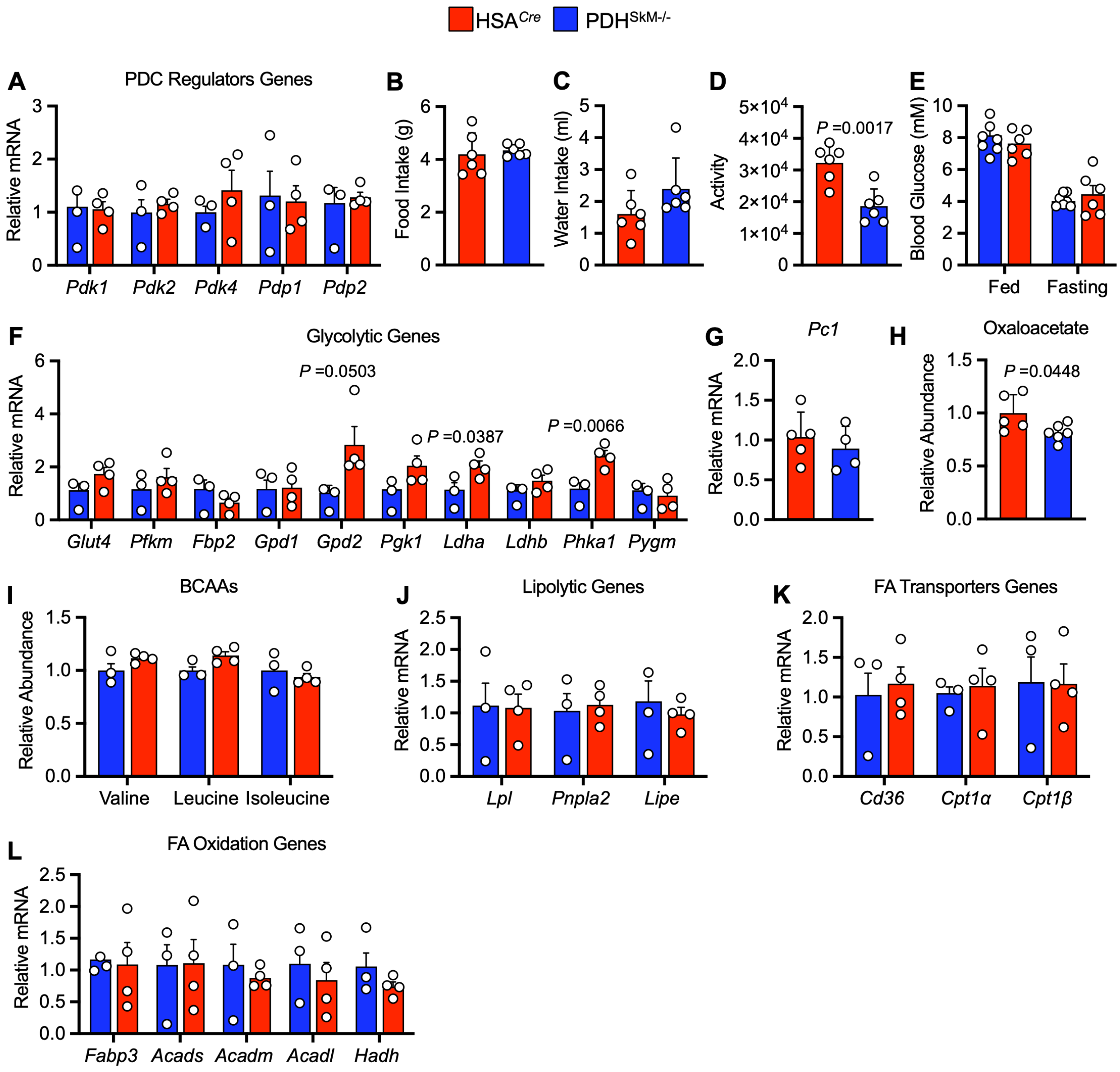

(**A**) mRNA expression of genes that regulate the PDH complex in gastrocnemius muscles collected from PDH$^{SkM-/-}$ and HSA^{Cre} littermates during the *ad libitum* state (*n*=4). Food intake (**B**), water intake (**C**), ambulatory activity (**D**), and blood glucose (**E**) were measured during the *ad libitum* and/or fasting state state (*n*=5-6). mRNA expression of glucose metabolism (**F**) and pyruvate carboxylase (*Pc*) 1 (**G**) genes, levels of oxaloacetate (**H**) and branched-chain amino acids (BCAAs) (**I**), and mRNA expression of lipolytic (**J**), fatty acid (FA) transporters (**K**) and oxidation (**L**) genes were evaluated in gastrocnemius muscles of PDH$^{SkM-/-}$ and HSA^{Cre} mice (*n*=4-5). Values are presented as means ± SD. *P* values were determined using an unpaired two-tailed Student's *t*-test.

Supporting Figure S2.

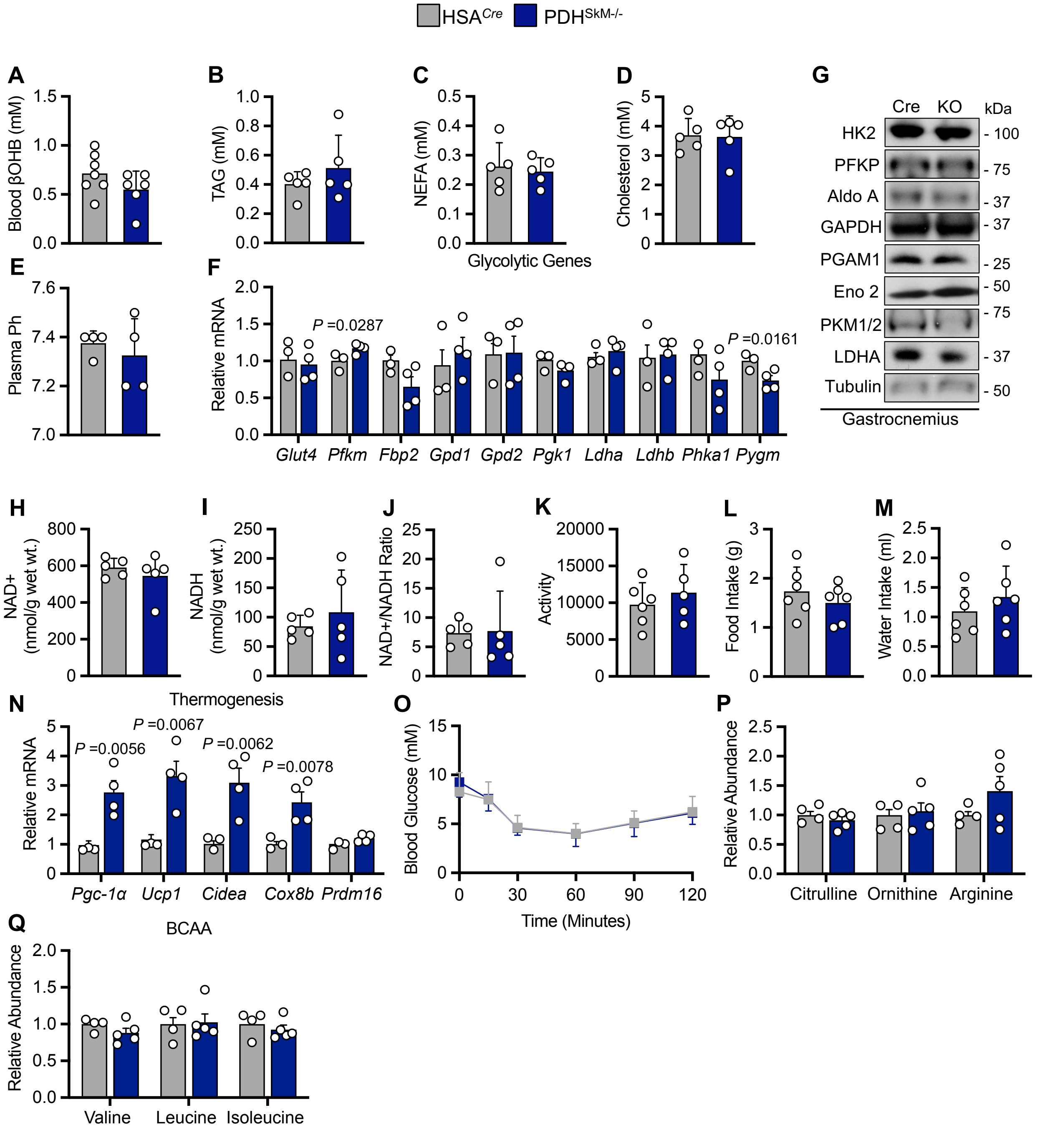

Levels of circulating β-hydroxybutyrate (βOHB) (**A**), triacylglycerol (TAG) (**B**), non-esterified fatty acids (NEFA) (**C**), cholesterol (**D**), and pH (**E**) were measured in obese PDH$^{SkM-/-}$ and HSA^{Cre} mice (*n*=4-7). mRNA (**F**) and protein (**G**) expression of glucose metabolism regulators, NAD+ levels (**H**), NADH level (**I**), and NAD^{+}/NADH ratio (**J**) were evaluated in gastrocnemius muscles of obese PDH$^{SkM-/-}$ and HSA^{Cre}

mice (*n*=4-5). Ambulatory activity (**K**), food intake (**L**), and water intake (**M**) were measured for 24-hr after one day in the metabolic cages (*n*=5-6). mRNA expression of thermogenesis-related genes in adipose tissues (**N**), insulin tolerance (**O**), and levels of citrulline/ornithine/arginine (**P**) and branched-chain amino acids (BCAAs) (**Q**) were measured in gastrocnemius muscles of obese PDH$^{SkM-/-}$ and HSA^{Cre} mice (*n*=4-7). *P* values were determined using an unpaired two-tailed Student's *t*-test or one-way repeated measures ANOVA followed by a Bonferroni *post-hoc* analysis.

Supporting Table S1.

Gene	Forward	Reverse
**PDH Complex Genes**		
Pdk1	GGACTTCGGGTCAGTGAATGC	TCCTGAGAAGATTGTCGGGGA
Pdk2	AGGGGCACCCAAGTACATC	TGCCGGAGGAAAGTGAATGAC
Pdk4	AGGGAGGTCGAGCTGTTCTC	GGAGTGTTCACTAAGCGGTCA
Pdp1	CTGCTGTTCACCACCATACA	GAAGCGTATCTCCTTCCTTGAG
Pdp2	ACTGTGTCCTACTGGATCTTCAA	CAGGTTCCTACTCGTGGCA
**Glycolytic Genes**		
Glut4	TTCATTGTCGGCATGGGTTT	ACGGCAAATAGAAGGAAGACGTA
Pfkm	CGTGGGAGAGCGTGTCTATGA	CTCGCTCTCGGAAGTCCTTG
Fbp2	GGTTCCATGGTGGCTGATGT	GGCCACAGGATTGCATTCAT
Gpd1	CCCATGAGCGTGCTGATG	GTGATGCGAAAGTTGGGTGTCT
Gpd2	CACGCACCATCCTATTCC	GACATCCCCTCTTCTCACTTC
Pgk1	GCTGTTCTCCTCTTCCTCATC	CCTTTGGTTGTTTGTTATCTGG
Ldha	TGCCTACGAGGTGATCAAGCT	GCACCCGCCTAAGGTTCTTC
Ldhb	AGTCTCCCGTGCATCCTCAA	AGGGTGTCCGCACTCTTCCT
Phka1	CATCTCTGCGCCTCTACCGTA	TGCTCATGTCGCCTTCACTG
Pygm	CTCCTCAACTGCCTGCACATC	ACCTATTGGGCTCCCTTTTGAT
**Anaplerotic Genes**		
Gpt1	GGAAGGTGCTAACTCTGGATAC	GGCACGGATAACCTCAGTAAA
Gpt2	AACTGTATCCGTGAAGATGTGGC	CAGGTAAATGTTGTCTGGGTCTG
Pc1	TGGGTTCCTCTCAGAGCGAG	GTCTCCCATCTTGCGGACC
Me2	GGCTAAGAGCTGTTACCACTCC	CGTAAACGCCATTCCCTTGTT
Me3	GAGATCGTGCGTCGCTTTGA	CGCAGCTCGACATAATGCTC
**Glutaminolysis Genes**		
Slc1a5	TGGGACCTCTTCCAGTTCCG	GGACTGCTGGCTGAGCTGTG
Gls	GCTGTGCTCTATTGAAGTGA	GCAAACTGCCCTGAGAAGTC

Got1	CTCCTCCGGTTCTGGTCTTT	CCCCAAGAACTAGGCGAGAA
Got2	CAGCCGAGATGTCTTTCTGC	GGACACTCTGCTCTGGGATT
Gdh1	GAGATGTCCTGGATCGCTGA	GGCCCACATTACCAAATCCC
Gs	ACAGCGACATGTACCTCCAT	CTGCTCCATTCCAAACCAGG
**Lipolytic Genes**		
Lpl	GGGAGTTTGGCTCCAGAGTTT	TGTGTCTTCAGGGGTCCTTAG
Pnpla2	GGATGGCGGCATTTCAGACA	CAAAGGGTTGGGTTGGTTCAG
Lipe	CCAGCCTGAGGGCTTACTG	CTCCATTGACTGTGACATCTCG
**FA Transporters Genes**		
Cd36	ATGGGCTGTGATCGGAACTG	GTCTTCCCAATAAGCATGTCTCC
Cpt1α	GAGAAATACCCTGACTATGTG	TGTGAGTCTGTCTCAGGGCTAG
Cpt1β	GCACACCAGGCAGTAGCTTT	CAGGAGTTGATTCCAGACAGGTA
**FA Oxidation Genes**		
Fabp3	ACCTGGAAGCTAGTGGACAG	TGATGGTAGTAGGCTTGGTCAT
Acads	TGGCGACGGTTACACACTG	GTAGGCCAGGTAATCCAAGCC
Acadm	GCTGGAGACATTGCCAATCA	GGCGTCCCTCATCAGCTTCT
Acadl	TCTTTTCCTCGGAGCATGACA	GACCTCTCTACTCACTTCTCCAG
Hadh	TTGCCAGCAACACGTCTTCTT	GAGGCCAGCAAATCGGTCTT
**Thermogenesis Genes**		
Pgc-1α	AGACAAATGTGCTTCGAAAAAGAA	GAAGAGATAAAGTTGTTGGTTTGGC
Ucp1	AAGCTGTGCGATGTCCATGT	AAGCCACAAACCCTTTGAAAA
Cidea	GGTTCAAGGCCGTGTTAAGG	CGTCATCTGTGCAGCATAGG
Cox8b	TGTGGGGATCTCAGCCATAGT	AGTGGGCTAAGACCCATCCTG
Prdm16	GCACGGTGAAGCCATTCATATG	TCGGCGTGCATCCGCTTGTG
**Other**		
Atf4	AAGGAGGAAGACACTCCCTCT	CAGGTGGGTCATAAGGTTTGG
